# Supplementary material for: Multimodal deep learning to predict prognosis in adult and pediatric brain tumors
Source: Commun Med (Lond). 2023 Mar 29;3:44. doi: 10.1038/s43856-023-00276-y (PMC10060397; doi:10.1038/s43856-023-00276-y)
Supplement: Supplementary file 2 — Supplementary Information [file 43856_2023_276_MOESM2_ESM.pdf]

Multimodal deep learning to predict prognosis in adult and pediatric brain tumors

Sandra Steyaert[1,*], Yeping Lina Qiu[1,2,*], Yuanning Zheng[1], Pritam Mukherjee[1], Hannes Vogel[3] and Olivier Gevaert[1,4**]

1) Stanford Center for Biomedical Informatics Research (BMIR), Department of Medicine, Stanford University, Stanford, CA, USA.
2) Department of Electrical Engineering, Stanford University, Stanford, CA, USA.
3) Department of Pathology, Stanford University, Stanford, CA, USA.
4) Department of Biomedical Data Science, Stanford University, Stanford, CA, USA.

* These authors contributed equally
** To whom correspondence should be addressed: ogevaert@stanford.edu,
ORCID: https://orcid.org/0000-0002-9965-5466

Supplementary Information

Supplementary Figures

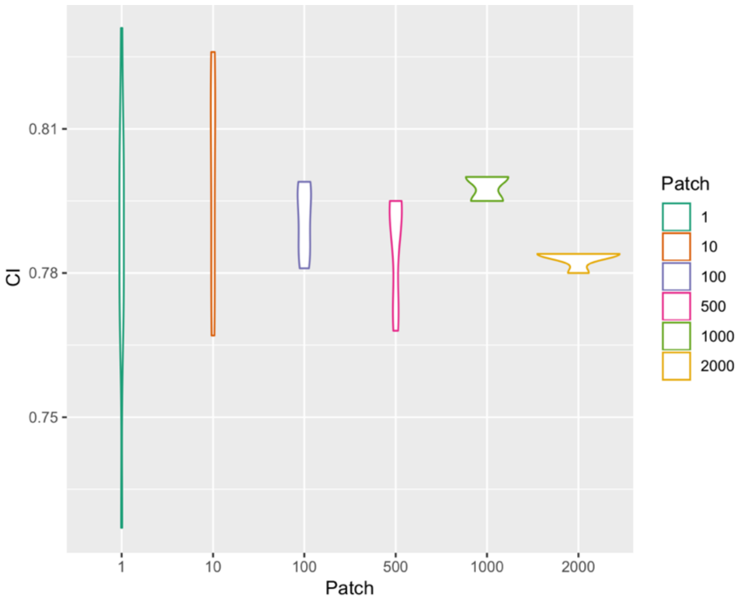

Supplementary Figure S1: Average Concordance Index (CI) for 10 training runs using either 1, 10, 100, 500, 1000 or 2000 patches per whole slide image (WSI). Indicated CI are the scores of the models on the test set (N=156).

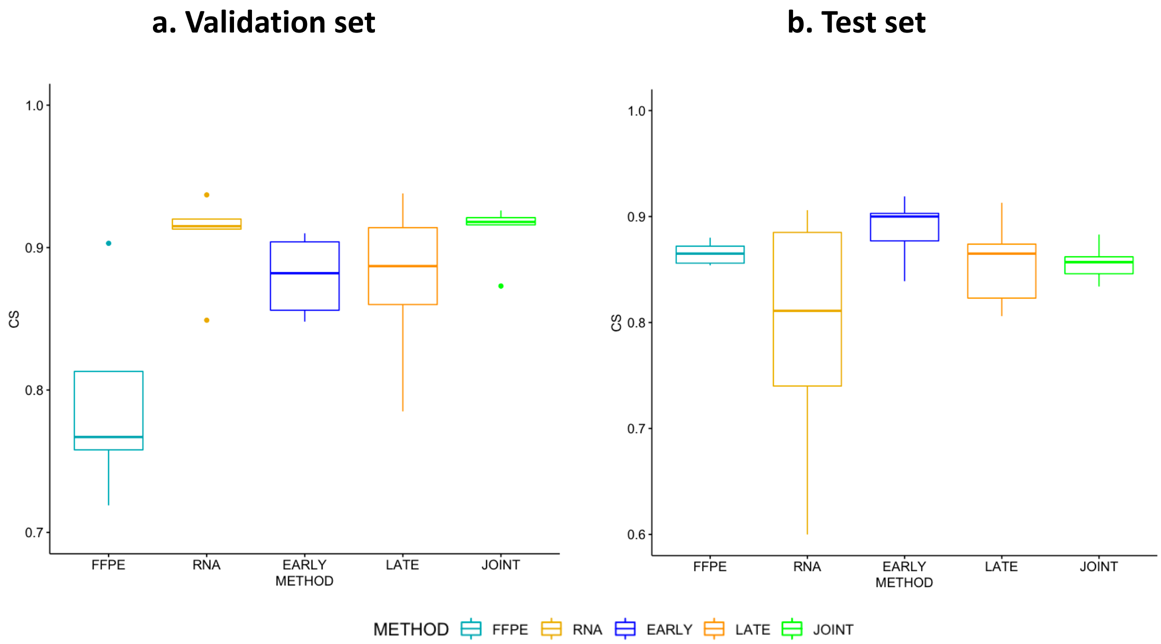

Supplementary Figure S2: Boxplots of model performance for each model strategy on the pediatric glioma cohort. a Composite Score **(**CS) distribution on cross validation (CV) validation sets (N=32). **b** CS distribution of each CV fold on the test set (N=39).

a. RNA only model

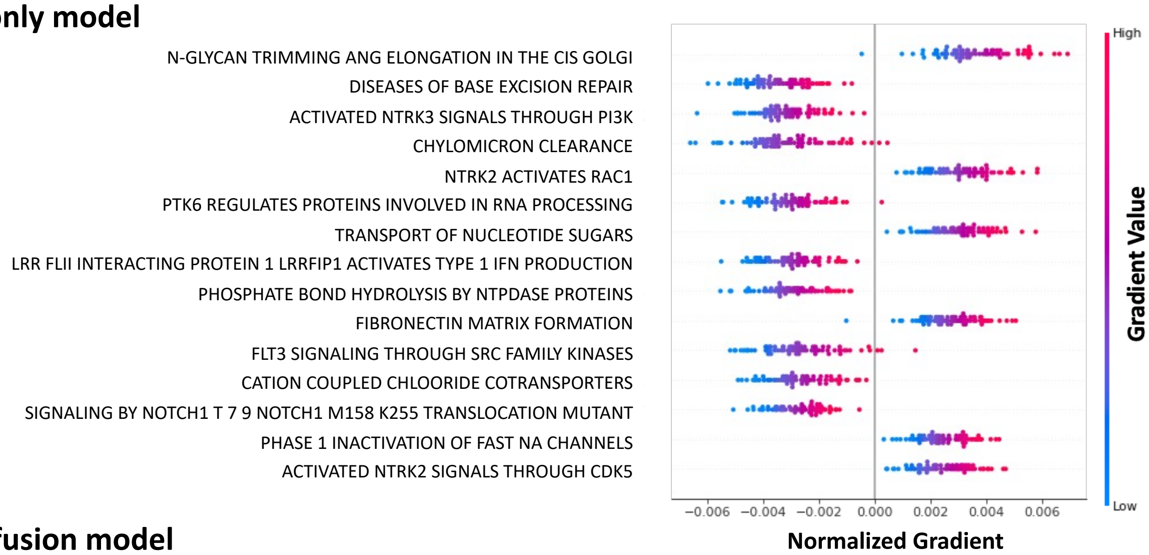

b. Joint fusion model

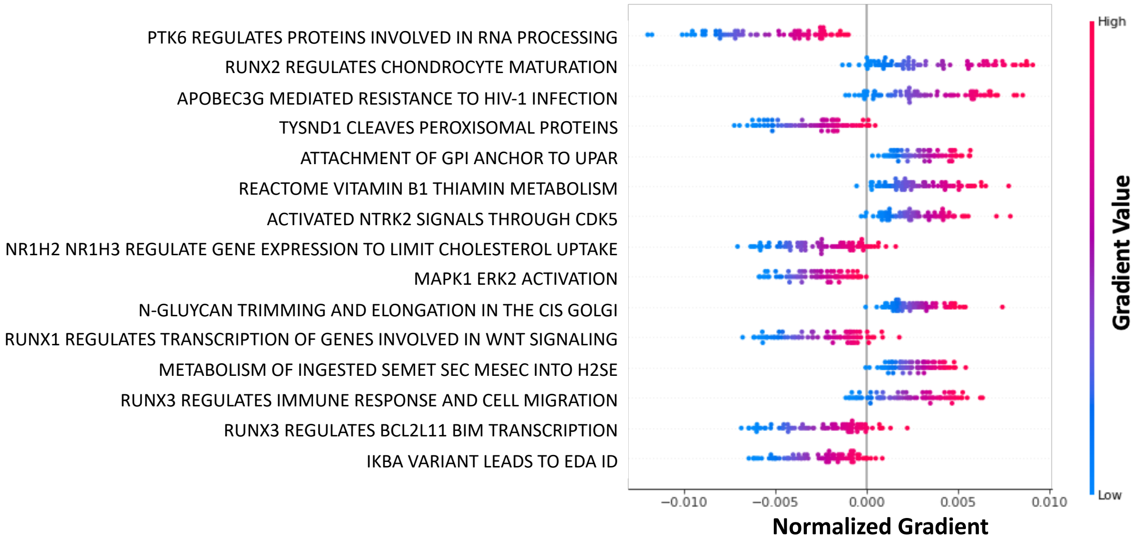

Supplementary Figure S3: Visualization of pathway importance with respect to survival predictions for the adult The Cancer Genome Atlas (TCGA) cohort. Pathways are ranked from top to bottom based on the sum of the absolute gradients across all samples. Negative gradients contribute to a lower risk score, while positive gradients lead to a higher risk score. **a** Top 15 pathways of the unimodal gene expression model (RNA only). **b** Top 15 pathways of the multimodal joint fusion model (histopathology + RNA data).

Supplementary Tables

Supplementary Table S1: Concordance Index scores (CI) of survival predictions on adult and pediatric glioma cohorts using two single modality models (FFPE & RNA), and three data fusion methods (early, late & joint). The first two data columns show the results (mean and standard deviation (stdev)) on the cross validation (CV) training and validation sets (10-fold CV for adult and 5-fold CV for pediatric cohort). The last column shows the result of the final model on the test set.

Cohort	Strategy	Model	Training set CV Mean(CI) ± *stdev*	Validation set CV Mean(CI) ± *stdev*	Test set CI
ADULT	Single modality	FFPE	0.841 *± 0.021*	0.764 *± 0.027*	0.768
		RNA	0.818 *± 0.032*	0.754 *± 0.055*	0.710
	Multimodal	Early Fusion	0.841 *± 0.011*	0.789 *± 0.020*	0.786
		Late Fusion	0.865 *± 0.015*	0.797 *± 0.019*	0.778
		Joint Fusion	0.846 *± 0.035*	0.779 *± 0.027*	0.788
PEDIATRIC	Single modality	FFPE	0.889 *± 0.011*	0.795 *± 0.097*	0.803
		RNA	0.899 *± 0.065*	0.934 *± 0.050*	0.860
	Multimodal	Early Fusion	0.912 *± 0.027*	0.896 *± 0.059*	0.908
		Late Fusion	0.931 *± 0.029*	0.905 *± 0.045*	0.947
		Joint Fusion	0.994 *± 0.001*	0.929 *± 0.026*	0.912

Supplementary Table S2: Integrated Brier Scores (IBS) of survival predictions on adult and pediatric glioma cohorts using two single modality models (FFPE & RNA), and three data fusion methods (early, late & joint). The first two data columns show the results (mean and standard deviation (stdev)) on the cross validation (CV) training and validation sets (10-fold CV for adult and 5-fold CV for pediatric cohort). The last column shows the result of the final model on the test set.

**Cohort**	**Strategy**	**Model**	**Training set CV Mean(IBS) ± *stdev***	**Validation set CV Mean(IBS) ± *stdev***	**Test set IBS**
**ADULT**	Single modality	FFPE	0.092 *± 0.011*	0.150 *± 0.015*	0.158
		RNA	0.096 *± 0.012*	0.135 *± 0.025*	0.151
	Multimodal	Early Fusion	0.089 *± 0.006*	0.127 *± 0.029*	0.115
		Late Fusion	0.082 *± 0.008*	0.132 *± 0.025*	0.134
		Joint Fusion	0.094 *± 0.017*	0.130 *± 0.023*	0.144
**PEDIATRIC**	Single modality	FFPE	0.089 *± 0.016*	0.212 *± o.088*	0.094
		RNA	0.099 *± 0.048*	0.121 *± 0.039*	0.159
	Multimodal	Early Fusion	0.121 *± 0.047*	0.136 ± 0.033	0.070
		Late Fusion	0.072 *± 0.038*	0.151 *± 0.084*	0.121
		Joint Fusion	0.031 *± 0.010*	0.108 *± 0.032*	0.189

Supplementary Table S3: Distribution of different prognostic subtypes of adult glioma based on genetic and diagnostic markers in the poor survival (PS) and good survival (GS) groups of each model. Values indicate the number of samples of the test set classified by each model in the two risk groups. IDHmut/wt = *IDH1* mutant/wildtype; (no)codel = (no) 1p/19q codeletion. For some samples, the genetic subtype was not known (NA).

Cohort	Group	Subtype	FFPE (PS / GS)	RNA (PS / GS)	Early Fusion (PS / GS)	Late Fusion (PS / GS)	Joint Fusion (PS / GS)
**ADULT test set**	Diagnostic	Glioblastoma	68 / 6	56 / 18	66 / 8	64 / 10	64 / 10
		Astrocytoma anaplastic	2 / 22	14 / 10	10 / 14	12 / 12	11 / 13
		Astrocytoma NOS	1 / 6	1 / 6	0 / 7	0 / 7	0 / 7
		Oligodendroglioma anaplastic	2 / 5	3 / 4	2 / 5	2 / 5	2 / 5
		Oligodendroglioma NOS	3 / 16	0 / 19	0 / 19	0 / 19	0 / 19
		Mixed Glioma	2 / 23	4 / 21	0 / 25	0 / 25	1 / 24
	Genetic	IDHmut-codel	6 / 14	1 / 19	0 / 20	0 / 20	0 / 20
		IDHmut-nocodel	2 / 41	2 / 41	0 / 43	0 / 43	1 / 42
		IDHwt	50 / 20	59 / 11	58 / 12	60 / 10	60 / 10
		NA	20 / 3	16 / 7	20 / 3	18 / 5	19 / 4

Supplementary Table S4: P-values of Kruskal-Wallis test and post-hoc Dunn Tests (with Benjamini-Hochberg P-value correction). Multiple pairwise comparisons of Composite Score (CS) on cross validation (CV) validation sets (N=63) (top) and CS distribution of each CV fold on the test set (N=156) for each model strategy (bottom). (*P-value < 0.05, **P-value < 0.01 and ***P-value < 0.005)

	**Kruskal-Wallis P-value = 0.028* (df=4, chi-squared=10.843)**				
Validation sets	***Post-hoc Dunn-test***	**FFPE**	**RNA**	**Early Fusion**	**Late Fusion**
	**RNA**	0.418	*NA*	*NA*	*NA*
	**Early Fusion**	**0.044***	0.056	*NA*	*NA*
	**Late Fusion**	0.059	0.052	0.425	*NA*
	**Joint Fusion**	0.076	0.098	0.410	0.326
	**Kruskal-Wallis P-value = 2.855e-07*** (df=4, chi-squared=36.038)**				
Test set	***Post-hoc Dunn-test***	**FFPE**	**RNA**	**Early Fusion**	**Late Fusion**
	**RNA**	**0.013***	*NA*	*NA*	*NA*
	**Early Fusion**	**0.001*****	**< 2.2e-16*****	*NA*	*NA*
	**Late Fusion**	0.064	**0.0001*****	0.065	*NA*
	**Joint Fusion**	0.245	**0.002*****	**0.008****	0.192

Supplementary Table S5: Concordance Index scores (CI) of the developed models on CPTAC, ependymoma and medulloblastoma cohorts. CPTAC: validation of adult glioma models on external cohort. Ependymoma and medulloblastoma: transfer learning of pediatric glioma models on pediatric brain tumor cohorts from a different origin with less samples.

**Strategy**	**Model**	**CPTAC CI**	**Ependymoma CI**	**Medulloblastoma CI**
Single modality	FFPE	0.563	0.519	0.495
	RNA	0.563	0.630	0.617
Multimodal	Early Fusion	0.593	0.588	0.637
	Late Fusion	0.588	0.569	0.609
	Joint Fusion	0.571	0.615	0.696
