# Supplementary material for: Multimodal deep learning to predict prognosis in adult and pediatric brain tumors
Source: Commun Med (Lond). 2023 Mar 29;3:44. doi: 10.1038/s43856-023-00276-y (PMC10060397; doi:10.1038/s43856-023-00276-y)
Supplement: Supplementary file 5 — Reporting Summary [file 43856_2023_276_MOESM5_ESM.pdf]

nature portfolio

Corresponding author(s): Olivier Gevaert (COMMSMED-21-0602B)

Last updated by author(s): Feb 15, 2023

Reporting Summary

Nature Portfolio wishes to improve the reproducibility of the work that we publish. This form provides structure for consistency and transparency in reporting. For further information on Nature Portfolio policies, see our Editorial Policies and the Editorial Policy Checklist.

Statistics

For all statistical analyses, confirm that the following items are present in the figure legend, table legend, main text, or Methods section.

n/a	Confirmed	
☐	☒	The exact sample size (*n*) for each experimental group/condition, given as a discrete number and unit of measurement
☒	☐	A statement on whether measurements were taken from distinct samples or whether the same sample was measured repeatedly
☐	☒	The statistical test(s) used AND whether they are one- or two-sided<br>*Only common tests should be described solely by name; describe more complex techniques in the Methods section.*
☐	☒	A description of all covariates tested
☐	☒	A description of any assumptions or corrections, such as tests of normality and adjustment for multiple comparisons
☐	☒	A full description of the statistical parameters including central tendency (e.g. means) or other basic estimates (e.g. regression coefficient) AND variation (e.g. standard deviation) or associated estimates of uncertainty (e.g. confidence intervals)
☐	☒	For null hypothesis testing, the test statistic (e.g. *F*, *t*, *r*) with confidence intervals, effect sizes, degrees of freedom and *P* value noted<br>*Give P values as exact values whenever suitable.*
☒	☐	For Bayesian analysis, information on the choice of priors and Markov chain Monte Carlo settings
☒	☐	For hierarchical and complex designs, identification of the appropriate level for tests and full reporting of outcomes
☒	☐	Estimates of effect sizes (e.g. Cohen's *d*, Pearson's *r*), indicating how they were calculated

Our web collection on statistics for biologists contains articles on many of the points above.

Software and code

Policy information about availability of computer code

Data collection	No software was used to collect the data. Data was immediately downloaded from their respective data portals (see data availability statement)
Data analysis	The source code of the models used to perform the results and analyses presented in this manuscript are available on GitHub at https://github.com/gevaertlab/MultiModalBrainSurvival. All analysis were performed using python v3.7.13 and R version 4.1.2. Model development was done using Pytorch v1.5.0. The Combat-Seq package (https://github.com/zhangyuqing/ComBat-seq) was used to account for any batch effects between RNAseq and microarray data. Integrated Brier Score values were calculated with the “survcomp” R package. Concordance indexes were calculated with the lifelines (https://github.com/CamDavidsonPilon/lifelines) survival analysis python package.

For manuscripts utilizing custom algorithms or software that are central to the research but not yet described in published literature, software must be made available to editors and reviewers. We strongly encourage code deposition in a community repository (e.g. GitHub). See the Nature Portfolio guidelines for submitting code & software for further information.

Data

Policy information about availability of data

All manuscripts must include a data availability statement. This statement should provide the following information, where applicable:

- Accession codes, unique identifiers, or web links for publicly available datasets
- A description of any restrictions on data availability
- For clinical datasets or third party data, please ensure that the statement adheres to our policy

Datasets analyzed during the current study were derived from their respective data portals: (i) Adult glioma cohort from The Cancer Genome Atlas (TCGA) available via the GDC data portal (https://portal.gdc.cancer.gov/repository), (ii) Pediatric brain tumor cohort from the Pediatric brain Tumor Atlas (PBTA) available through the Gabriella Miller Kids First Data Resource Portal (KF-DRC, https://kidsfirstdrc.org) and (iii) Adult glioblastoma cohort from the National Cancer Institute's Clinical Proteomic Tumor Analysis Consortium (CPTAC) with RNA-seq data available at the GDC data portal (https://portal.gdc.cancer.gov/repository) and the pathology images at The Cancer Imaging Archive (TCIA) Portal (https://www.cancerimagingarchive.net/datascope/cptac/home/). The source code of the models used to perform the results and analyses presented in this manuscript are available on GitHub at https://github.com/gevaertlab/MultiModalBrainSurvival.

Human research participants

Policy information about studies involving human research participants and Sex and Gender in Research.

Reporting on sex and gender	Reported findings are not gender-specific but apply for both Females and Males. Gender was considered in the study setup to split the data in a stratified fashion with equal contribution to each. Gender information was made available as metadata when downloading the samples from their respective repositories. All patients involved in the databases have given ethical approval.
Population characteristics	The used adult brain cancer cohorts consisted of patients diagnosed with glioma (low-grade glioma or glioblastoma). Only patients having both histopathology data (FFPE) and expression data (RNAseq or microarray) were selected. For the pediatric cohort, patients of 4 brain tumor subtypes were selected: low-grade gliom, astrocytoma, ependymoma and medulloblastoma and only patients having both histopathology images and RNA-seq data were kept. For all cohorts, age, gender, age at index and vital status were available as metadata.
Recruitment	For this study no specific patient recruitment was done but open-source brain tumor data from three sources was used: (i) Adult cohort from TCGA, (ii) Pediatric cohort from the PBTA available through the Gabriella Miller Kids First Data Resource Portal and (iii) Adult patients from the National Cancer Institute's Clinical Proteomic Tumor Analysis Consortium Glioblastoma Multiforme (CPTAC-GBM) cohort.
Ethics oversight	The study was conducted in accordance with the Declaration of Helsinki (as revised in 2013). Our present study was based upon data from three open-source multi-institutional efforts; (i) The Cancer Genome Atlas (TCGA, https://www.cancer.gov/tcga), (ii) Gabriella Miller Kids First Pediatric Research Program (Kids First, https://commonfund.nih.gov/kidsfirst/overview) and (iii) National Cancer Institute's Clinical Proteomic Tumor Analysis Consortium (CPTAC, https://proteomics.cancer.gov/programs/cptac). The patients involved in the database have given ethical approval. All samples have been collected and utilized following strict human subjects' protection guidelines, informed consent, and IRB review of protocols. The relevant data is accessible for free and can be downloaded for research and to publish relevant articles.

Note that full information on the approval of the study protocol must also be provided in the manuscript.

Field-specific reporting

Please select the one below that is the best fit for your research. If you are not sure, read the appropriate sections before making your selection.

☒ Life sciences ☐ Behavioural & social sciences ☐ Ecological, evolutionary & environmental sciences

For a reference copy of the document with all sections, see nature.com/documents/nr-reporting-summary-flat.pdf

Life sciences study design

All studies must disclose on these points even when the disclosure is negative.

Sample size	The used adult brain cancer cohort consisted of a glioma cohort with LGG and GBM samples. Only samples having both histopathology (FFPE) data and expression data were selected. In the TCGA repository, for a total of 426 LGG samples and 158 GBM samples also RNA-seq data was available. For GBM, also samples with microarray expression data were included thereby adding 199 patients. The final adult TCGA glioma dataset consisted of 783 samples, with 426 LGG samples and 357 GBM samples. For the CPTAC GBM cohort there were 97 patients having both histopathology images and RNA-seq data.<br><br>Also for the pediatric cohort, samples having both histopathological images and RNA-seq were selected (N=305). LGG and astrocytoma samples were combined in a pediatric glioma group (N=198). For ependymoma and medulloblastoma there were 47 and 60 patient samples, respectively.

Data exclusions	For the samples described above, no data was excluded.
Replication	A 10-fold stratified cross validation (CV) was performed on the training set of the adult cohort, while a 5-fold stratified CV strategy was chosen for the pediatric glioma cohort (since this cohort contained less samples). A separate test set (see below) was left out during model training and only used for calculating model robustness and performance of each fold.
Randomization	For each cohort, samples were shuffled into a training and test set at a 80/20 ratio with stratification on age, gender, tumor grade (high/low) and survival time. Similary, for the cross-validation, for each fold, each cohort, samples were shuffled into a training and validation set with stratification on age, gender, tumor grade (high/low) and survival time.
Blinding	Blinding was not relevant to the study.

Reporting for specific materials, systems and methods

We require information from authors about some types of materials, experimental systems and methods used in many studies. Here, indicate whether each material, system or method listed is relevant to your study. If you are not sure if a list item applies to your research, read the appropriate section before selecting a response.

Materials & experimental systems

n/a	Involved in the study
☒	☐ Antibodies
☒	☐ Eukaryotic cell lines
☒	☐ Palaeontology and archaeology
☒	☐ Animals and other organisms
☒	☐ Clinical data
☒	☐ Dual use research of concern

Methods

n/a	Involved in the study
☒	☐ ChIP-seq
☒	☐ Flow cytometry
☒	☐ MRI-based neuroimaging
